# Heterogeneous network embedding enabling accurate disease association predictions

**DOI:** 10.1186/s12920-019-0623-3

**Published:** 2019-12-23

**Authors:** Yun Xiong, Mengjie Guo, Lu Ruan, Xiangnan Kong, Chunlei Tang, Yangyong Zhu, Wei Wang

**Affiliations:** 1 Shanghai Key Laboratory of Data Science, School of Computer Science, Fudan University, Shanghai, China; 2 Shanghai Institute for Advance Communication and Data Science, Fudan University, Shanghai, China; 3 Department of Computer Science, Worcester Polytechnic Institute, Worcester, USA; 4 Brigham and Women’s Hospital, Harvard Medical School, Boston, USA; 5 Department of Computer Science, Scalable Analytics Institute (ScAi), University of California, Los Angeles, USA

**Keywords:** Network embedding, Heterogeneous network, Disease association prediction

## Abstract

**Background:**

It is significant to identificate complex biological mechanisms of various diseases in biomedical research. Recently, the growing generation of tremendous amount of data in genomics, epigenomics, metagenomics, proteomics, metabolomics, nutriomics, etc., has resulted in the rise of systematic biological means of exploring complex diseases. However, the disparity between the production of the multiple data and our capability of analyzing data has been broaden gradually. Furthermore, we observe that networks can represent many of the above-mentioned data, and founded on the vector representations learned by network embedding methods, entities which are in close proximity but at present do not actually possess direct links are very likely to be related, therefore they are promising candidate subjects for biological investigation.

**Results:**

We incorporate six public biological databases to construct a heterogeneous biological network containing three categories of entities (i.e., genes, diseases, miRNAs) and multiple types of edges (i.e., the known relationships). To tackle the inherent heterogeneity, we develop a heterogeneous network embedding model for mapping the network into a low dimensional vector space in which the relationships between entities are preserved well. And in order to assess the effectiveness of our method, we conduct gene-disease as well as miRNA-disease associations predictions, results of which show the superiority of our novel method over several state-of-the-arts. Furthermore, many associations predicted by our method are verified in the latest real-world dataset.

**Conclusions:**

We propose a novel heterogeneous network embedding method which can adequately take advantage of the abundant contextual information and structures of heterogeneous network. Moreover, we illustrate the performance of the proposed method on directing studies in biology, which can assist in identifying new hypotheses in biological investigation.

## Background

Correctly predicting new disease associations with other biological entities(e.g. genes, miRNAs) has long been an important goal in biomedical research. With the emergence of large-scale disease-related association datasets in biology, scientists can leverage statistical and machine learning methods to assist in achieving this goal. Singh-Blom et al. [1] propose a supervised machine learning method that uses a biased support vector machine where the features are derived from walks in a heterogeneous gene-trait network to predict gene-disease associations. Chen et al. [2] introduce random walk with restart method to prioritize the candidate disease for miRNAs. Zeng et al. assess the correlation between nodes by the HeteSim score [3] for the purpose of predicting disease-gene associations [4] and disease-miRNA associations [5]. However, these methods only extract simple features from datasets and there still exist many challenges as discussed below.

Recent technological advances have enabled researchers to produce and investigate an enormous quantity of data to illustrate the underlying biological mechanisms of complicated diseases [6] better. Consequently, many large databases have been developed to preserve and organize the accumulated data, which were generated and conserved by extensive collaboration. For instance, the DisGeNET database [7] collects a comprehensive catalogue of genes and variants involved in human diseases from various expert-curated repositories [1, 4, 8, 9], and the miRNet database [10] integrates data from eleven disease-miRNA databases [5, 11]. In addition, almost all of these datasets supply perceived and/or inferred knowledge about relations between diseases and other biological entities. For instance, the MISIM database [12] preserves a miRNA similarity network; the Human Reference Protein Database (HPRD) [13] keeps a network of protein-protein interaction; the MimMiner [14] offers a similarity network of diseases. Capturing the complicated biological relationships among data requires a systematic method to ponder these multifaceted data simultaneously, involving genes [15], proteins [16], miRNAs [17], drugs [18], side-effects [19] and so on. It may shed light not only on understanding the mechanisms in complex diseases, but also on identifying new biological hypotheses to direct future explorations and researches. Although several big consortia such as ENCODE and GTEx have made remarkable progress, we discover a growing disparity between our capabilities of producing data and the capabilities of integrating, investigating, and explaining data. The majority of recent researches typically concentrate on data produced in the environment managed by themselves or by their colleagues, in order to make sure that data is produced in homogeneous conditions thus can be compared directly. Accordingly, data produced from previous researches and the inferred knowledge preserved in available repositories are still widely underutilized. And it is unpractical to fully utilize such enormous amount of data to conduct biological experiments due to high expenses. Moreover, heterogeneity of data types, experimental environments and experimental technologies is a primary challenge. Consequently, we design a network-based analytic model to tackle these challenges.

**Fig. 1:**
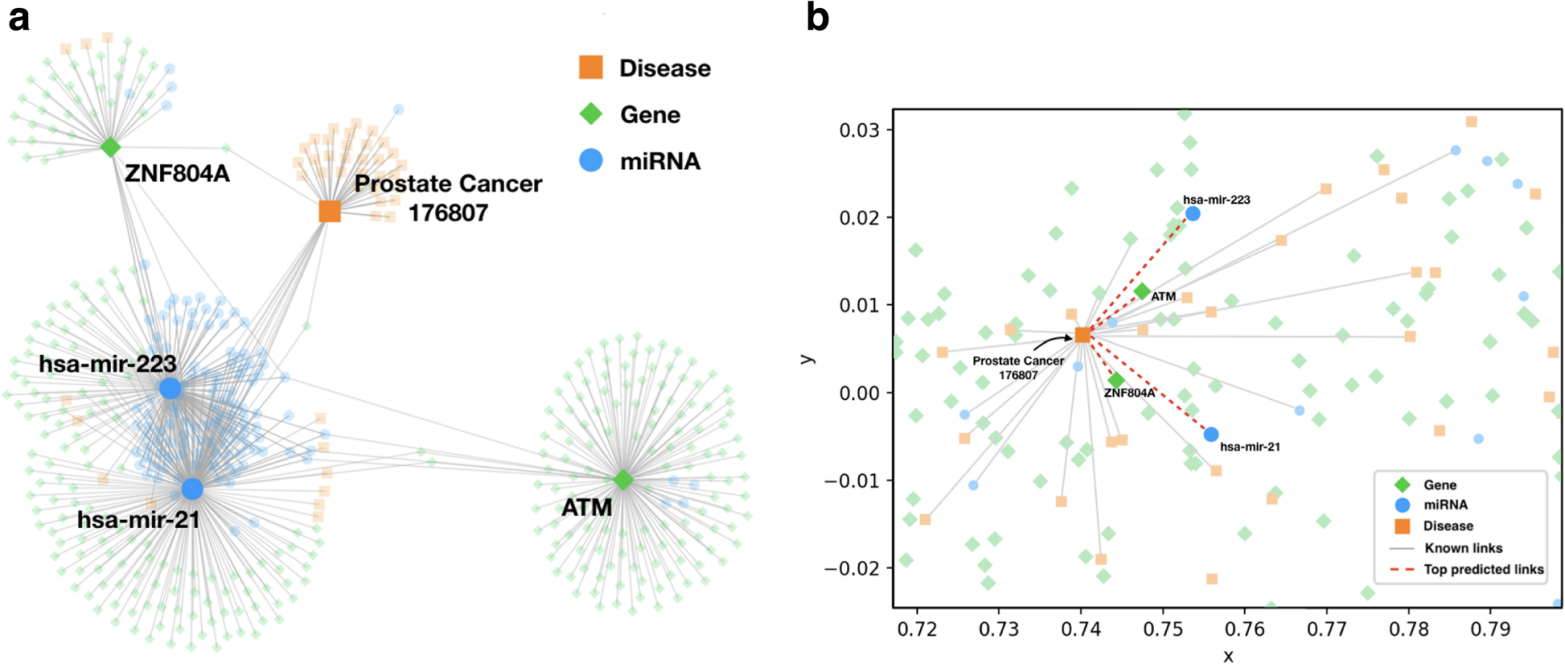
An illustrative example of heterogeneous biological network embedding. The left figure demonstrates one disease, two miRNAs, two genes, and their known links which are denoted by solid edges. The right figure presents their projection to the two-dimensional space of a small region around the disease by employing network embedding. The four red dashed links denote the top predicted links utilizing our model. **a** Sub-network Relation Visualization. **b** Network Embedding Visualization

We are motivated by the discovery that networks in which nodes indicate entities such as proteins, diseases and edges indicate relationships between these entities can represent a majority of the above-mentioned data. Because there exist various types of entities, the relationships may be likewise of various types (e.g. protein-protein interaction, disease-miRNA association). Besides, nodes and edges may have auxiliary attributes such as node features and link weights which further describe the characteristics of the entities and relations. For the sake of making full use of the knowledge carried by the constructed network, we apply the network embedding method [20, 21] which has successfully presented its effect in exploring and discovering relationships between persons within social networks. Network embedding maps the network data into a continuous low-dimensional feature space which preserves the vertex content, side information and topological structure, especially existent relationships. Every entity (e.g., protein, disease) is embeded to a low-dimensional vector and mapped to a point in the vector space. And if the relationship between two entities is stronger, they are closer in the vector space. Figure 1a demonstrates a sub-network which contains one disease (i.e., prostate cancer), two miRNAs (i.e., hsa-mir-223, hsa-mir-21) and two genes (i.e., ZNF804A, ATM), as well as their existent links to other diseases, miRNAs, and genes. Figure 1b displays a projection of a tiny region around prostate cancer in the two-dimensional embedding space where genes and miRNAs which are actually connected to prostate cancer are distributed in the proximity of this disease. The four red dashed edges denote the top two miRNAs and two genes which don’t possess direct links but have great possibility of connecting to prostate cancer in the prediction of our model.

Representation learning for the aforementioned heterogeneous networks confronts some challenges. Nodes in a network may represent entities of vastly different characteristics. And edges may represent disparate relationships, and each of which may be of various weight or other attribute. Conventional network embedding methods [20–22] are focused on homogeneous networks and based on skip-gram [23] model to learn the topological structures and other latent attributes of networks. Recently, deep neural networks have been introduced into homogeneous network embedding, [24–26] utilize graph convolution networks (GCNs) which generalize the operation of convolution [27] from traditional data (images or grids) to graph data and learn the connectivity structures from the adjacency matrices of graphs. There are also several existing works on heterogeneous network embedding [28–31]. Translation-based models [28, 29] learn representations of entities (nodes) and relationships (links) in knowledge graphs which can be regarded as heterogeneous networks, but these models only preserve the local structure by interpreting relations as translations and ignore the link weights in the network. Another kind of methods [30, 31], which decompose a heterogeneous network to a set of subgraphs and then perform embedding individually, ignore the different semantics of relationships in each subgraph and only capture the aggregated information of relationships by combining embedding of each subgraph. Moreover, [32, 33] consider the distinctive characteristics of relations (or entities) in the heterogeneous network, but [32] only projects different kinds of nodes (i.e., image and text) into the same vector space by neural networks which ignores the semantic information interpreting contextual properties in the heterogeneous network, and [33] distinguishes heterogeneous relations into two categories by structure-related measures and utilizes two different embedding models for each but there exist relations which can not be well distinguished by the structure-related measures in various heterogeneous networks. Although [34, 35] introduce meta path [35] to capture the rich semantic information in heterogeneous network, they don’t present how to select proper meta path in different networks especially in the biological network.

Another challenge is the scalability of the network embedding method. Heterogeneous networks provide a large amount of information about node relations. However, it is non-trivial to capture a large number of heterogeneous relationships. And it is impossible to list all neighbor nodes under different relations when the network scales up. Therefore, we need a scalable method to capture such rich relations efficiently.

To overcome the aforementioned challenges, in this paper, we propose HeteWalk, which is based on meta path [35*] controlled random walk* for representations learning in heterogeneous networks. Besides, we consider the edge weights during the representation learning and provide a *random walk-based measure* to assist in selecting meta path. We utilize the meta paths to capture abundant semantic information involved in the heterogeneous network. And the random walk procedure, which has shown the scalability in exploring large-scale networks [20], is controlled by not only the meta paths but also link weights on our network. In the embedding vector space, entities which are close to each other but at present do not possess direct links(edges) are probably connected and thus are significant subjects in future biological study.

In order to demonstrate the effectiveness of our method, we construct a heterogeneous network of diseases, genes and miRNAs using data from six real-world datasets and conduct two disease-related prediction tasks including disease-gene association prediction and disease-miRNA association prediction. Then we compare the proposed method with several advanced disease association prediction methods as well as some typical network embedding methods. The experimental results show the superiority of our proposed method. Moreover, we perceive that embracing additional datasets to train our method will promote the accuracy of the predicted results at all time. Furthermore, substantial associations we predict are verified by the latest miRNet dataset [10], which demonstrates our method can effectively provide guidance to discover new disease-related associations in biological studies.

## Methods

### Network construction

The accumulated biological data has been preserved and organized in massive databases, nevertheless, only a fraction of data generated from previous studies has been utilized. And the heterogeneity in data types, experimental technologies as well as experimental settings remains a vital challenge. We demonstrate the construction of a weighted heterogeneous network by integrating data from various databases in this section.

#### Datasets description

We utilize real-world data in six public sources to interpret the definition and effectiveness of the proposed method. These biological datasets offer the association networks and similarity networks between three types of entities which are diseases, miRNAs and genes. The detailed description of these biological networks are as follows:

- Gene (proteins) interaction network: We obtain 39,240 protein-protein interactions (PPI) from the Human Protein Reference Database (HPRD) [13] which was manually extracted from biological literature. For the pair of proteins with direct connections, their corresponding protein-coding genes are linked through an unweighted edge in the HPRD network and we set the weight as 1.0.
- miRNA similarity network: We acquire the similarities of miRNA functions from the MISIM databank [12], which provides the functional similarity of 271 miRNAs in pairs. The similarity score for each link which is calculated by the MISIM method ranges from 0 to 1.
- Disease phenotype similarity network: The similarities of human disease are extracted from the MimMiner [14], which utilizes a text-mining method for the classification of human diseases from the Online Mendelian Inheritance in Man (OMIM) database [36]. All links are associated with their own similarity scores ranging from 0 to 1 calculated by the MimMiner system.
- Gene-Disease association network: We extract this network from DisGeNET database [7], which incorporates gene-disease associations of humans from various professional databases. 19,714 entries whose disease phenotypes can be related to OMIM terms are used. Every association possess a score ranging from 0 to 1 in accordance with confidence, which is called DisGeNET score [7] with taking into account the number of sources supporting the association and the reliability of each of them.
- Gene-miRNA interaction network: The gene-miRNA interactions are provided by the miRTarBase database [37], which is gathered through manual survey of literature relevant to miRNAs’ functional studies. Reporter assay, western blot, microarray or next-generation sequencing experiments verify the collected interactions experimentally. At the step of network construction, We set the weights of 7269 interactions supported by strong experimental evidences (reporter assay or western blot) as 1, and set the weights of 13,990 interactions supported by weak experimental evidences (microarray or pSILAC) as 0.3. And the experimental evidence is justified by many crosslinking and immunoprecipitation sequencing (CLIP-seq) datasets which were generated by 21 independent studies [37].
- miRNA-Disease association network: Two datasets are combined to build this network. One dataset provides 242 miRNA-disease associations offered by Chen et al. 11]. The other is derived from the miRNet dataset [10], which contains substantial confirmed associations of miRNA-disease incorporated from HMDD [38], miR2Disease [39], and Phenomir [40], from which we extract the records whose disease names are able to connect with their OMIM ids then we obtain 666 disease-miRNA associations. And 878 miRNA-disease associations which totally includes 267 miRNAs and 59 diseases are acquired after deleting duplicated records. Because the associations have been validated at a high level of confidence, we determine all the weights as 1.0.

#### Weighted heterogeneous network construction

**Fig. 2:**
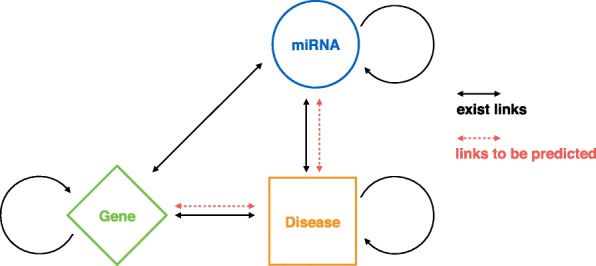
Network schema of constructed heterogeneous network. The solid black lines denote the links observed from the real-world network, and the red dashed lines denote the links we want to predict

We build a weighted heterogeneous network by joining the six above-mentioned networks entirely through shared nodes. And in these networks, genes are denoted by their gene symbols in HPRD [13], miRNAs are denoted through their names while disease phenotypes are denoted through their respective OMIM ids [36]. We summarize each sub-network of the constructed heterogeneous network in Table 1. The Fig. 2 presents the network schema, which comprises three types of nodes, in which rhombuses denote genes, circles denote miRNAs while squares denote diseases. The solid black lines indicate the existing connections in the aforementioned network, and the red dashed lines indicate the links to be predicted, involving disease-gene associations as well as disease-miRNA associations.

**Table 1:** Description of each sub-network of the constructed heterogeneous network

Network		Number of links	Weight	Source
Gene (proteins) interaction network	G - G	39,240	1	HPRD [13]
microRNA similarity network	M - M	56,289	0 to 1	MISIM [12]
Disease phenotype similarity network	D - D	3,162,016	0 to 1	MimMiner [14]
Gene-Disease association network	G - D	19,714	0 to 1	DisGeNET [7]
Gene-miRNA interaction network	G - M	21,259	0.3 or 1	miRTarBase [37]
miRNA-Disease association network	M - D	878	1	Chen et al. [11] and miR2Disease [10]

The constructed heterogeneous network includes various types of entities as well as relationships(links) with different weights. But it is not appropriate to compare the weights of links in different types directly since they come from distinct datasets. For instance, if the link weight of *prostate cancer*(disease) and *hsa-mir-21*(miRNA) is lower than that of *prostate cancer* and *ATM* (gene), it may not suggest that *hsa-mir-21* holds weaker association with *prostate cancer* than *ATM*. Consequently, in terms of a heterogeneous network, we need to map the network into a vector space where similarities and interactions between entities of different types can be numerically measured and predicted.

### HeteWalk

HeteWalk is a network embedding method which can generate a low dimensional representation vector for every entity in the heterogeneous network, which captures the structural and semantic information, especially the existent relationships. A critical inspiration for our method is that diseases (or genes, miRNAs) which are in close proximity to each other in the network have higher potential to be associated. For instance, a miRNA which plays an important part in a disease may be possible to play a similar part in a similar disease. This intuition equips us to make unknown disease-related link predictions founded upon the existent edges.

#### Network embedding

Lately, several network embedding methods [20,21] have presented competitive performance in various tasks such as node classification, link prediction and clustering. For the purpose of learning effective node representations for a network, we would like to maximize the probability of a node occurring given that its connected nodes (i.e., those with direct links) have occurred [20*,*^22^]. Given a node *v*_*i*_ and the set of connected nodes *N*(*v*_*i*_), we want to maximize the conditional probability of observing *N*(*v*_*i*_) for the node *v*_*i*_. The probability of observing each node is assumed to be independent of another, we want to maximize the following objective function:

1 $$  \prod_{v_{i} \in \mathcal{V}}{\Pr(N(v_{i})|v_{i})} = \prod_{v_{i} \in \mathcal{V}}\prod_{v_{j} \in N(v_{i})}{\Pr(v_{j}|v_{i})}  $$

We define the conditional probability as follows:

2 $$  \Pr(v_{j}|v_{i}) = \frac{e^{\vec{x_{i}}\cdot\vec{x_{j}}}}{\sum_{k \in V}e^{\vec{x_{i}}\cdot\vec{x_{k}}}},  $$

where *V* is the set of whole nodes in the network. $\vec {x_{i}}$ is the embedding vector for node *v*_*i*_ while $\vec {x_{j}}$ is the embedding vector for node *v*_*j*_. The whole vectors of nodes are latent *d*-dimensional vectors via learning based on the objective function.

The majority of existent network embedding methods focus on homogeneous networks where the types of whole nodes and edges are identical. In the setting of our constructed network, a disease node is possible to link to other diseases, genes or miRNAs, which are not in a single type. In order to fully capture the abundant contextual information and semantic properties of a node in such a complicated network, we would better to go further than direct-linked nodes. For instance, if a gene and a disease are related via a path involving several links such as $Gene\xrightarrow {similar\, with}Gene\xrightarrow {associated\, with}Disease$ or $Gene\xrightarrow {associated\,with}miRNA\xrightarrow {similar\, with}miRNA\xrightarrow {associated\, with}Disease$, they may be related as well. Next, we present how to take advantage of such paths in the heterogeneous network embedding.

#### Meta path-controlled random walk

A *meta path*$\mathcal {P}$ is a path which describes a composite relation between two objects, and we use the form of $\mathcal {A}_{1}\rightarrow \mathcal {A}_{2}\rightarrow \cdots \rightarrow \mathcal {A}_{m}$ to denote a meta path, where $\mathcal {A}_{i}$ denotes a type of nodes (e.g., disease, gene) [35]. We can use different meta-paths to classify multiple relationships which two nodes may possess in a heterogeneous network. For instance, the meta-path $Gene\xrightarrow {assoc}Disease$ represents a direct gene-disease connection; the meta path $Gene\xrightarrow {assoc}miRNA\xrightarrow {assoc}Disease$ presents a relationship that a gene and a disease are connected to a common miRNA; and the meta path $Gene\xrightarrow {sim}Gene\xrightarrow {assoc}Disease$ represents that a gene is similar to another gene which is associated with a disease. It’s obvious that semantics underneath these meta paths are different.

Meta-path is a powerful approach to describe indirect relationships among specific types of nodes. The quantity of different meta-paths increases exponentially with the amount of types in entity and relation and also the length of meta paths, supplying fruitful semantic information interpreting contextual characteristics of the network. Furthermore, in order to consider the link weights at the same time, we apply a *meta path-controlled random walk* to search the associated entities for each meta path. A meta path indicates what type of neighbor node should be visited at each step, then the link weights determine the probability to be chosen for each node with the determined type. We will demonstrate how to construct and select meta-paths in “Meta-path selection” and “Experimental settings” sections. Starting at node *v*_*i*_ with type *A*_*k*_, given a meta path $\mathcal {P}$ = $\mathcal {A}_{1}\rightarrow \mathcal {A}_{2}\rightarrow \cdots \rightarrow \mathcal {A}_{m}$, the random walk procedure will only visit a connected node in type $\mathcal {A}_{k+1}$ on the next step. If there are several nodes in type $\mathcal {A}_{k+1}$, we randomly choose a node with a probability proportional to the weight of link. If the link weight is higher, the node is more likely to be selected. For each node *v*_*i*_ with type $\mathcal {A}_{k}$, we define its transition probability to another node *v*_*j*_ as:

3 $$  {}\Pr(v_{j}|v_{i};\mathcal{P}) \!=\left\{ \begin{array}{rcl} \frac{w_{ij}}{\sum_{\phi(v_{k}) = \mathcal{A}_{k+1}} w_{ik}} & & (v_{i}, v_{j}) \in E, \phi(v_{j}) = \mathcal{A}_{k+1}\\ 0 & &(v_{i}, v_{j}) \in E, \phi(v_{j}) \neq \mathcal{A}_{k+1} \\ 0 & &(v_{i}, v_{j}) \notin E \end{array} \right.  $$

where *E* denotes the edge set of the network, *ϕ*(*v*_*i*_) denotes the node type while *w*_*ij*_ indicates the link weight for *v*_*i*_ and *v*_*j*_. The random walk procedure will create a node sequence starting from each node guided by a meta path. For the purpose of producing adequate node sequences, we repeat the random walk procedure which starts from every node.

#### Meta-path selection

Though a variety of meta paths can be defined by combining different node types, too many meta paths are redundant and may lead to low-efficiency. Besides, some meta paths may carry misleading information, which can be interference to the tasks [41]. So it’s significant to select proper meta path(s). Here we propose a random walk-based measure to assist in selecting meta path.

During a random walk, we want to visit as many nodes as possible to capture more characteristics of the network. Given a candidate set of meta-paths, for each meta path, the random walk procedure controlled by the meta path is repeated *m* times for each node, then we count the amount of nodes whose visited times are no larger than *m* and we call these nodes as *isolated walking nodes*. For a meta path $\mathcal {P}$, the random walks is repeated *m* times for every node in the network, then the *random walk-based measure* is the defined as the count of *isolated walking nodes*:

4 $$  C(\mathcal{P};m)=\sum_{v_{i} \in V} I(t_{i} \leq m),  $$

where *I* is the indicator function. *V* is the set of whole nodes in the network and *t*_*i*_ is the visited times of node *v*_*i*_ by random walks. The value of *random walk-based measure* for the meta path is smaller, random walks controlled by the meta path will visit more nodes and capture more attributes of the network thus this meta path is better to be selected.

#### Negative sampling

After obtaining a set of node sequences, our next step is to learn the vector representations for each node. As illustrated in Eq. (1), we aim at maximizing the probability of each node occurring given its linked nodes. That is, for nodes occurring in the identical node sequence, their node representations will be updated to maximize Eq. (1). There exist a massive amount of node pairs in all node sequences, thus it is very costly to compute Eq. (1). Enlightened by the optimization in word embedding methods, we employ negative sampling [23] to approximate:

5 $$ {}\log\Pr(v_{j}|v_{i}) \!= \! \log\sigma(\vec{x_{i}}\cdot\vec{x_{j}})+ \! \sum_{n=1}^{K}{\mathbb{E}_{v_{n}\sim NEG(v_{j})}\log\sigma(-\vec{x_{i}}\cdot\vec{x_{n}})},  $$

where $\sigma (x) = \frac {1}{1 + e^{-x}}$ is the sigmoid function, and *NEG*(*v*_*j*_) is the distribution to sample a negative node *v*_*n*_. Besides, *K* is the number of negative samples.

We randomly choose *K* negative node pairs (*v*_*i*_,*v*_*N*_) in which *v*_*N*_≠*v*_*j*_ and *ϕ*(*v*_*N*_)=*ϕ*(*v*_*j*_) for each node pair (*v*_*i*_,*v*_*j*_) occurring in the same node sequence. We train the model via maximizing the score of positive sample (*v*_*i*_,*v*_*j*_) while minimizing the scores of all negative samples (*v*_*i*_,*v*_*N*_). For instance, given a node sequence (*Disease*1,*Disease*2,*Gene*1) created by the meta path $Disease\xrightarrow {sim}Disease\xrightarrow {assoc}Gene$, we obtain 3 positive node pairs (*Disease*_1_,*Disease*_2_),(*Disease*_1_,*Gene*_1_) and (*Disease*_2_,*Gene*_1_). Take (*Disease*_1_,*Gene*_1_) as an instance, subsequently, *K* nodes of gene type are randomly selected, which are symbolized by $Gene_{N_{1}}, \cdots, Gene_{N_{K}}$, where $Gene_{N_{i}} \neq Gene_{1}$. The positive sample (*Disease*_1_,*Gene*_1_) and *K* negative samples $\phantom {\dot {i}\!}(Disease_{1}, Gene_{N_{i}})$ are fed into the model at the same time and we use Stochastic Gradient Descent (SGD) [42] to update their corresponding representation vectors based on Eq. (5).

#### Disease associations prediction

All types of nodes (diseases, genes and miRNAs) in our heterogeneous network are mapped to the common vector space after network embedding. Then the cosine distance between node vectors are used to assess their relationships. As to the prediction of disease-related associations, if a disease and a gene/miRNA without direct link in the network but are in proximity to each other in the projected vector space, it is very likely for them to be associated so they are promising to study in biological investigation.

## Results and discussion

### Comparison to baselines

We compared our method HeteWalk with several state-of-the-art baselines so as to measure its performance. We partitioned these baseline methods into two groups. One group consist of CATAPULT [1], HSMP and HSSVM [4*,*^5^], which are conventional statistical and machine learning methods without network embedding and specially designed to identify a particular type of associations (i.e., disease-miRNA or disease-gene). These methods were operated on our constructed heterogeneous network. CATAPULT utilizes features extracted from paths with different lengths based on a biased support vector machine. And HSMP and HSSVM evaluate the relevance between nodes utilizing the HeteSim score [3], which judges the accessibility between two nodes along a given path. HSMP joins HeteSim scores in multiple paths to a constant which inhibits the long paths’ contributions, and HSSVM integrates HeteSim scores utilizing a supervised machine learning method.

Methods in the other group are representative network embedding methods including DeepWalk [20], LINE [21], DGI [26], TransE [28*] and AspEm [*31]. DeepWalk is a typical homogeneous network embedding method, which uses a vanilla random walk procedure and learns representations of vertices by treating walks as sentences. LINE, which also ignores the heterogeneous information, preserves both first-order and second-order proximities and is suitable for arbitrary large-scale information networks such as our constructed network. DGI is the latest homogeneous network embedding method using established graph convolutional network (GCN) [24] architectures as far as we know. TransE, which models relationships as translations in the embedding space of entities, is a typical knowledge graph embedding method where the knowledge graph can be regarded as a heterogeneous network. AspEm learns embedding by aspects, with each aspect representing one underlying semantic facet of the heterogeneous network.

HeteWalk applies *meta path-controlled random walks* for heterogeneous network embedding. We utilize the embeded vectors of nodes for prediction of entities (e.g., genes, miRNAs) which have great chances to be associated with diseases.

### Experimental settings

We experimentally evaluated the effectiveness of predicting two types of association including gene-disease association and miRNA-disease association. The vector dimension is set to 128, the number of walks per node and per meta path to 10, while the size of negative samples is set to 5 following the common practice in network embedding [21*,*^31^]. In addition, we set the margin to be 1 and the dissimilarity measure to be L2 for TransE based on the best validation performance. Besides, we utilized one-hot representation of each node as node features and a weighted adjacency matrix extracted from our constructed network in DGI as input. And for AspEm, since nodes may appear different times in the selected set of representative aspects (e.g., one node may occur in two aspects, while another may occur in only one), and the dimension of the vector learned from each aspect was the same, we filled zeros for those vectors whose dimensions were below 128. We demonstrated in “Parameter analysis” section that the performance is insensitive to the settings on the vector dimension and the number of walks.

In the progress of constructing meta path, all non-redundant meta paths related to target entity types were extracted separately in the first step. After that, redundant meta paths were formed by combining two or more. Since long meta paths are useless to capture the link structure [35],only short meta paths with restricted length were extracted. Then we obtained the candidate set of meta paths. Moreover, we selected meta path from the candidates by utilizing the *random walk-based measure* in which the number of random walks is 10, the same with original experimental set. The meta paths we extracted and their corresponding values of the measure are shown in Table 2. We can see that the measure of meta path “GGD” is smallest with the value 8658 in gene-disease association prediction, which is the same with the selected meta path according to our experience (best test results by cross validation on each meta-path). But for miRNA-disease association prediction, the smallest measure value belongs to the meta path “MGGD”, different from our experience, in which the performance of meta path “MMDD” was best (“G” denotes gene, “M” denotes miRNA and “D” denotes disease). This is mainly because the number of miRNA-Disease interaction edges is far less than other types of edges in the network as we can observe from Table 1. Additionally, the measure value of “MMDD” is smallest among meta paths with only two node types (i.e. miRNA and disease). We can select the meta path not only by experience, but also use the *random walk-based measure*, which can be regarded as the a auxiliary approach to reduce the time cost on experiments. We utilized the meta-path “GGD” for gene-disease association prediction and “MMDD” for miRNA-disease association prediction in subsequent experiments. CATAPULT, HSMP, HSSVM, and our HeteWalk used the same meta paths.

**Table 2:** Meta paths and their *random walk-based measures* between gene-disease and miRNA-disease

	With 2 types of nodes	Measure	With 3 types of nodes	Measure
gene-disease	Gene$\xrightarrow {sim} \text {Gene} \xrightarrow {assoc} \text {Disease} \xrightarrow {sim}$ Disease	9364	Gene$\xrightarrow {assoc}\text {miRNA}\xrightarrow {assoc}$Disease	16103
	Gene$\xrightarrow {sim}\text {Gene}\xrightarrow {assoc}$Disease	8658	Gene$\xrightarrow {sim}\text {Gene}\xrightarrow {assoc}\text {miRNA}\xrightarrow {assoc}$Disease	10465
	Gene$\xrightarrow {assoc}\text {Disease}\xrightarrow {sim}$Disease	14422	Gene$\xrightarrow {assoc}\text {miRNA}\xrightarrow {sim}\text {miRNA}\xrightarrow {assoc}$Disease	16084
	Gene$\xrightarrow {assoc}\text {Disease}\xrightarrow {assoc}\text {Gene}\xrightarrow {assoc}$Disease	10184	Gene$\xrightarrow {assoc}\text {miRNA}\xrightarrow {assoc}\text {Disease}\xrightarrow {sim}$Disease	16460
miRNA-disease	miRNA$\xrightarrow {sim}\text {miRNA}\xrightarrow {assoc}\text {Disease}\xrightarrow {sim}$Disease	19381	miRNA$\xrightarrow {assoc}\text {Gene}\xrightarrow {assoc}$Disease	14820
	miRNA$\xrightarrow {sim}\text {miRNA}\xrightarrow {assoc}$Disease	21323	miRNA$\xrightarrow {assoc}\text {Gene}\xrightarrow {sim}\text {Gene}\xrightarrow {assoc}$Disease	10011
	miRNA$\xrightarrow {assoc}\text {Disease}\xrightarrow {sim}$Disease	19540	miRNA$\xrightarrow {sim}\text {miRNA}\xrightarrow {assoc}\text {Gene}\xrightarrow {assoc}$Disease	15481
	miRNA$\xrightarrow {assoc}\text {Disease}\xrightarrow {assoc}\text {miRNA}\xrightarrow {assoc}$Disease	21335	miRNA$\xrightarrow {assoc}\text {Gene}\xrightarrow {assoc}\text {Disease}\xrightarrow {sim}$Disease	14626

### Effectiveness measurement

In each experiment, we randomly partitioned the known disease associations into 10 sets with same size, and we utilized a subset for training while the left for testing. As regards testing, in each experiment, the known associations were regarded as positive samples, randomly selecting the same amount of node pairs which have the same node types and no associations as negative samples, the cosine distance between the embedding vectors of the node pair in each sample was the predicted value. The proportion of training set varied from 50% to 90%. We repeated the experiments 10 times and reported the average Area under Receiver Operating Characteristic curve (AUROC) score for each training ratio. We demonstrate the results in Table 3 (gene-disease association prediction) and Table 4 (miRNA-disease association prediction).

**Table 3:** AUROC Score on Gene-Disease Association Prediction

Method/Training ratio	50%	60%	70%	80%	90%
CATAPULT	0.611	0.619	0.622	0.659	0.685
HSMP	0.621	0.625	0.679	0.708	0.747
HSSVM	0.609	0.653	0.693	0.734	0.779
DeepWalk	0.454	0.461	0.481	0.433	0.477
LINE(1st+2nd)	0.638	0.655	0.647	0.667	0.661
DGI	0.523	0.527	0.549	0.561	0.534
TransE	0.488	0.496	0.492	0.488	0.496
AspEm	**0.639**	0.667	0.659	0.657	0.681
HeteWalk	0.638	**0.674**	**0.723**	**0.759**	**0.798**

The best performance is in bold

**Table 4:** AUROC Score on miRNA-Disease Association Prediction

Method/Training ratio	50%	60%	70%	80%	90%
CATAPULT	0.811	0.833	0.843	0.867	0.877
HSMP	0.833	0.864	0.878	0.899	0.869
HSSVM	0.841	0.877	0.902	0.922	0.932
DeepWalk	0.498	0.511	0.534	0.611	0.677
LINE(1st+2nd)	0.780	0.795	0.829	0.813	0.804
DGI	0.501	0.483	0.496	0.516	0.512
TransE	0.473	0.477	0.481	0.469	0.464
AspEm	0.765	0.819	0.761	0.849	0.819
HeteWalk	**0.937**	**0.951**	**0.953**	**0.946**	**0.969**

The best performance is in bold

It is obvious that our method outperforms other methods in both disease association prediction tasks under entire training ratios except for the gene-disease association prediction with 50% training data in which the AUROC score of HeteWalk is 0.638, slightly inferior to the best score which is 0.639 achieved by AspEm. With more training data, the advantage of our method becomes more significant. In practice, the training ratio is almost always much bigger than 50%. For the miRNA-disease association prediction task, HeteWalk achieves a significantly excellent AUROC score 0.969 in 90% training ratio. However, the best score on the gene-disease prediction task is 0.798, because there exist relatively larger amount of candidate gene-disease associations.

HeteWalk demonstrates the superiority over heterogeneous network-based baselines, involving CATAPULT, HSMP, HSSVM, TransE,and AspEm. CATAPULT, HSMP, and HSSVM use the same set of meta paths with HeteWalk, but only simple features on accessibility between two nodes along path are extracted by them. By contrast, HeteWalk preserves existent relationships through maximizing the conditional probability of each node pair occurring given other pairs in a node sequence which is created based on the meta path. Though TransE considers the heterogeneity in node (entity) and edge (relation) types, it only preserves the local structures in the network represented by observed links and ignores link weights while our HeteWalk preserves global structures by *meta path-controlled random walks* in addition to the local structures and the selected nodes on random walk are determined by both link weight and meta path. AspEm learns embedding vectors from each aspect (selected subgraph) and then gets the final embedding for each node by concatenating the learned vectors from all aspects involving that node, so a problem occurs that not all embedding vectors are in the same vector space and some important information learned from the network may be lost after projecting all representation vectors to the same vector space.

The main reason why DeepWalk, LINE, DGI show poor performance is that they are specially designed for homogeneous networks. For DeepWalk, when selecting the next node to visit during a random walk, it ignores the differences between various types of relationships and treats all types of nodes equally. LINE, which preserves both local and global structures by first-order and second-order proximity, also ignores node and link types. DGI utilizes the weighted adjacency matrix as structure features which does not distinguish between different node and link types. As a result, it may be unlikely for the embedding methods mentioned above to successfully conserve the relationships between specific entities.

### Advantage of heterogeneity

We investigated the capability for each method to deal with heterogeneity and presented the advantage to incorporate various data sources. We constructed another two heterogeneous networks which only consist of two types of nodes. We solely joined G-G, G-D and D-D networks described in Table 1 for the gene-disease association prediction task. And only D-D, M-M, and D-M networks are used in the miRNA-disease association prediction task.

**Fig. 3:**
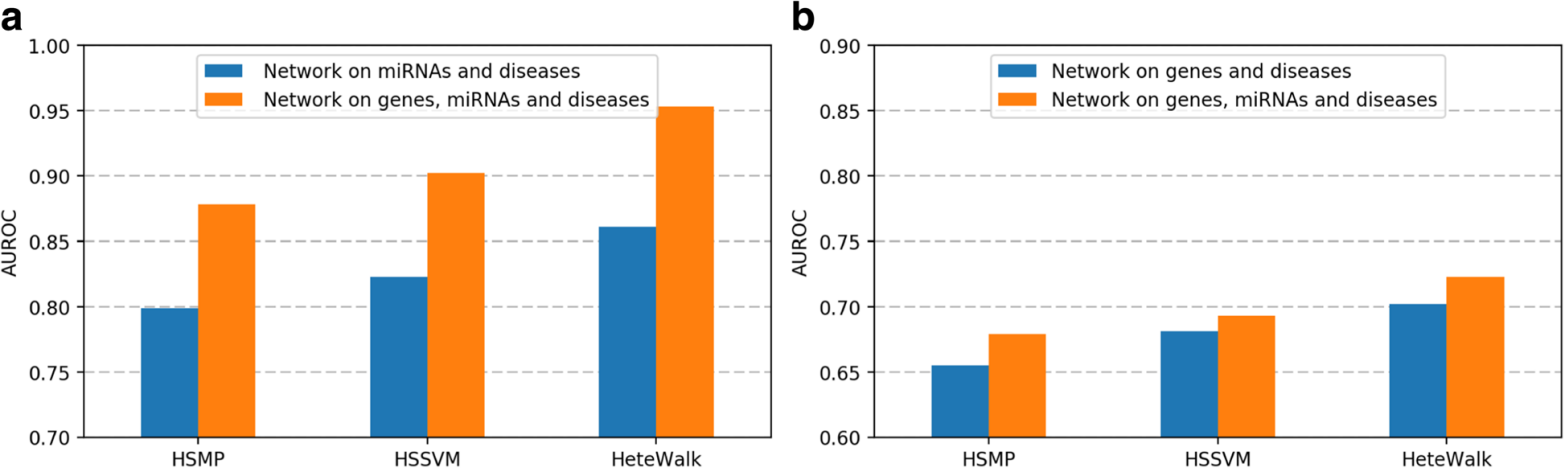
Performance on different networks. The left figure illustrates the AUROC score of miRNA-disease association predicted by two comparable methods and our method, in which the blue bar denotes the results on a sub-network only containing data in miRNA and disease types and the orange one is on the whole heterogeneous network. The right figure illustrates the score of gene-disease association prediction, in which the blue bar denotes the results on a sub-network only containing data in gene and disease types and the orange one is on the whole network. **a** miRNA-disease association prediction. **b** Gene-disease association prediction

We conducted 3-fold cross validation in the experiment, that is the known disease associations are divided into three parts with same size, and two parts are used to train and another to test each time. We compare the average score on two tasks for each method in Fig. 3. Conspicuous improvement is observed via combining networks to construct a bigger and more complex one, particularly in the miRNA-disease association prediction tasks. This may own to sparse relations between miRNAs and diseases, thus it is fairly unreliable to make predictions based on these relations alone. The gene-related data provide some information about indirect relations between miRNAs and diseases, which is possibly obtained via the meta paths. It demonstrates that potential knowledge of complicated diseases can be dug through integrating multifaceted data, which promote our prediction results to a greater extent. Alhough we have presented the effectiveness of HeteWalk on six databases, HeteWalk is actually able to incorporate any amount of data which could be represented by a network. The amount of types of node and link are not limited.

### Parameter analysis

**Fig. 4:**
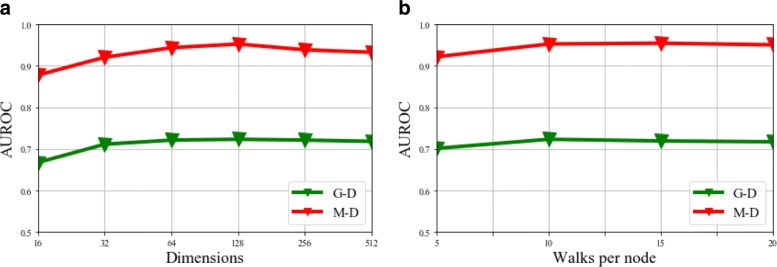
Parameter sensitivity. The green broken-line denotes the results on gene-disease association prediction, while the red broken-line denotes the results on miRNA-disease association prediction. **a** AUROC for different embedding dimensions. **b** AUROC for different number of walks

We explored the sensitivity of parameters in HeteWalk following the same setting as the 3-fold cross validation above-mentioned. We present the performance with various vector dimensions and various number of walks for each node in Fig. 4. We can find that the optimal performance is attained around 128 dimensions from Fig. 4a. Besides, we can observe the AUROC result remains almost steady when the amount of walks per node exceed 10 from Fig. 4b. Therefore, we set the vector dimensions as 128 and walks for each node as 10 in the experiment due to the performance and computational cost.

### Top-ranked predicted associations for specified diseases

The top-ranked gene/miRNA candidates for eight disease phenotypes predicted by HeteWalk are listed detailedly in Table 5, so as to investigate which may play a dominant part in a particular disease.

**Table 5:** Top 10 unknown disease-related associations predicted by HeteWalk

	Gene		miRNA		Gene		miRNA
Leukemia OMIM: 601626				Alzheimer disease OMIM: 104300			
2	TNF	3	hsa-mir-21	2	GRN	1	hsa-mir-223
4	APOE	4	hsa-mir-17	8	CHMP2B	2	hsa-mir-659
5	ATM	7	hsa-mir-146a	10	TNF	3	hsa-let-7c
6	PRRX1	8	hsa-mir-510	12	CEBPA	4	hsa-mir-21
7	CD81	10	hsa-mir-20b	13	ATM	5	hsa-mir-15a
8	USP8	11	hsa-mir-331	15	PPARG	6	hsa-mir-16-1
9	PPARG	12	hsa-mir-155	16	BCR	7	hsa-mir-17
10	IL1B	13	hsa-mir-143	17	ABL1	8	hsa-mir-155
11	SH2B1	14	hsa-mir-539	18	USP8	9	hsa-mir-510
12	IL6	15	hsa-mir-192	19	HNF1B	11	hsa-let-7a-1
Insulin resistance OMIM: 125853				Prostate cancer OMIM: 176807			
1	BCR	1	hsa-mir-659	1	ATM	1	hsa-mir-223
2	ABL1	2	hsa-mir-21	2	ZNF804A	2	hsa-mir-21
4	ARID3B	3	hsa-mir-223	3	BEND2	4	hsa-mir-144
8	MAST1	4	hsa-let-7c	4	TBP	5	hsa-mir-331
9	CEBPA	5	hsa-mir-16-1	5	PLTP	6	hsa-mir-17
11	CDH8	6	hsa-mir-15a	6	ELP5	8	hsa-mir-510
12	ZNF609	7	hsa-mir-17	7	KLHL35	10	hsa-mir-143
13	TBP	8	hsa-mir-155	8	ENTPD6	11	hsa-mir-20b
14	IL1RAPL1	9	hsa-mir-146a	9	RBP2	12	hsa-mir-425
15	ENTPD6	10	hsa-mir-510	10	U2AF2	14	hsa-let-7a-1
Schizophrenia OMIM: 181500				Breast cancer OMIM: 114480			
1	CEBPA	1	hsa-mir-21	1	PHKG1	2	hsa-let-7c
2	TNF	2	hsa-let-7c	2	FGF4	3	hsa-mir-223
3	EVPL	3	hsa-mir-223	3	CEBPA	4	hsa-mir-16-1
4	PPARG	4	hsa-mir-16-1	4	EVPL	7	hsa-mir-15a
5	AKT2	5	hsa-mir-15a	5	HAVCR1	10	hsa-mir-539
6	HAVCR1	6	hsa-mir-146a	6	BCR	12	hsa-mir-20b
7	PHKG1	7	hsa-mir-155	7	TBP	13	hsa-mir-484
8	APOE	8	hsa-mir-510	8	PPARG	14	hsa-mir-192
9	ENPP1	9	hsa-mir-17	9	CDH1	15	hsa-mir-93
10	FGF4	10	hsa-mir-20b	10	AKT2	16	hsa-mir-614
Gastric cancer OMIM: 137215				Colorectal cancer OMIM: 114500			
1	FTO	2	hsa-mir-146a	1	ESRRB	1	hsa-mir-146a
2	NTRK1	3	hsa-mir-155	2	COL3A1	2	hsa-mir-16-1
3	PCSK1	5	hsa-mir-539	3	GNA11	4	hsa-mir-155
4	MSH6	6	hsa-mir-484	4	GDF1	5	hsa-mir-20b
5	RAI1	7	hsa-let-7c	5	ZMPSTE24	6	hsa-mir-93
6	DICER1	8	hsa-mir-192	6	COL4A5	7	hsa-mir-192
7	DHH	9	hsa-mir-614	7	KIF11	8	hsa-mir-539
8	MC3R	10	hsa-mir-21	8	CLCN2	10	hsa-mir-181b-1
9	NOG	11	hsa-mir-181b-1	10	REST	11	hsa-mir-510
10	GDF1	12	hsa-mir-34b	11	SCN3B	12	hsa-mir-203a

For each disease, the top-ranked genes are in the left column while the top-ranked miRNAs are in the right. The numbers denote their original ranking before known associations are removed in the results

These candidates are ranked depending on their cosine distances to each selected disease. For the purpose of concision, the existent associations are not displayed here.

We discover that the existent associations are not always ranked high on the list, though the diseases possess many directly related genes and miRNAs in our real-world datasets. For instance, there exist 33 known genes associated with insulin resistance (125853) in the datasets, but only 5 of them are within the top-10 genes for this disease. This results from their relatively low link weights in our constructed network, which denotes a weak relation to insulin resistance. And in our method, several meta paths can extract the complex relationship with insulin resistance for genes without direct links, so these genes may distribute closer to the disease in the embedding space than some actually connected genes. Besides, there also exist many unknown associations with genes or miRNAs predicted for other diseases, which may assist biologists in identifying new disease relations.

### Validation and comparison of the top-ranked miRNA-disease associations prediction

To validate our approach, we manually checked the miRNA-disease associations predicted by our algorithm based on the miRNet dataset [10], which contains a massive collection of verified miRNA-disease associations from miR2Disease [39], HMMD [38] and Phenomir [40]. As each disease is represented by a disease name instead of its OMIM id, we only combined part of the records (666 of 19,342) to construct the heterogeneous network, the left of which were utilized to validate the top-ranked miRNA-disease associations predicted by our HeteWalk.

In the experiment, all datasets in Table 1 was utilized to generate the heterogeneous network and our method was applied to learn the representation vector for each node. Table 6 reports the top 10 diseases predicted to have associations with each of the four miRNAs (i.e., hsa-mir-21, hsa-let-7a-1, hsa-mir-125b-1 and hsa-mir-155), which possess the largest amount of verified records in the miRNet dataset. Among these predictions, we identified 8, 7, 6, and 7 confirmed associations for hsa-mir-21, hsa-let-7a-1, hsa-mir-125b-1 and hsa-mir-155, respectively, demonstrating the effectiveness of our methods.

**Table 6:** Top 10 diseases associated to the given miRNAs predicted by HeteWalk

Rank	Disease	Verified
hsa-mir-21		
3	188550 Nonmedullary Thyroid cancer 1	miR2Disease
5	608232 Chronic myeloid leukemia	PhenomiR
6	266600 Inflammatory bowel disease 1	HMDD
8	607464 Thyroid carcinoma	
9	273300 Male germ cell tumor	
10	151430 B-cell lymphoma 2	PhenomiR
11	155601 Cutaneous malignant melanoma	PhenomiR
12	145500 Hypertension	HMDD
13	256700 Neuroblastoma	HMDD
14	176807 Prostate cancer	PhenomiR, HMDD, miR2Disease
hsa-let-7a-1		
2	155255 Medulloblastoma	PhenomiR
4	176807 Prostate cancer	PhenomiR, HMDD, miR2Disease
6	256700 Neuroblastoma	PhenomiR
7	608232 Chronic myeloid leukemia	PhenomiR
9	151430 B-cell lymphoma 2	PhenomiR
10	150699 Uterine leiomyoma	
12	600634 Pituitary adenoma	miR2Disease
15	236000 Hodgkin lymphoma	PhenomiR, HMDD, miR2Disease
16	607464 Thyroid carcinoma	
18	226150 Enterocolitis	
hsa-mir-125b-1		
1	137800 Glioma susceptibility 1	miR2Disease
2	266600 Inflammatory bowel disease 1	
4	188550 Nonmedullary Thyroid cancer 1	HMDD
5	273300 Male germ cell tumor	
6	608232 Chronic myeloid leukemia	PhenomiR
7	155601 Cutaneous malignant melanoma	HMDD
9	145500 Hypertension	
10	181500 Schizophrenia	
11	151430 B-cell lymphoma 2	PhenomiR
13	260350 Pancreatic cancer	PhenomiR, HMDD, miR2Disease
hsa-mir-155		
2	188550 Nonmedullary Thyroid cancer 1	HMDD
3	273300 Male germ cell tumor	
4	137800 Glioma susceptibility 1	HMDD
6	155601 Cutaneous malignant melanoma	HMDD
7	608232 Chronic myeloid leukemia	PhenomiR
8	256700 Neuroblastoma	
10	601626 Acute myeloid leukemia	PhenomiR, HMDD
12	226150 Enterocolitis	
13	114500 Colorectal cancer	PhenomiR, HMDD
15	176807 Prostate cancer	PhenomiR

The first column shows the rankings of the predictions among all diseases, the second presents their diseases names and OMIM ids, and the third indicates whether the predicted associations are verified

The first column in Table 6 presents the rank of the corresponding predicted disease among all associated diseases, and their disease name as well as OMIM id are in column two. The last column indicates whether the predicted associations is verified in miRNet and, if so, the verification source is given. There are 7, 11, 4, and 6 known disease associations in the training set for hsa-mir-21, hsa-let-7a-1, hsa-mir-125b-1, and hsa-mir-155, respectively. We can find that some of the known associations which actually exist were not ranked highly. The reasons are two-fold. First, some of these associations possess relatively low weights, suggesting a weak relationship with the disease. Second, while some diseases and miRNAs do not currently possess direct links in the training data, they are well related to each other by several meta paths in the heterogeneous network. These diseases are therefore considered more associated to the miRNAs than those that are directly connected but with low link weights and are more likely to be predicted by HeteWalk.

The top 10 disease phenotypes for these four miRNAs predicted by alternative baselines (i.e., CATAPULT, HSMP and HSSVM) are listed in Tables 7, 8 and 9, with records verified by miRNet indicated in bold. We omit the known associations in these tables too and the first column indicates their original rankings. We compare them with the results predicted by HeteWalk.

**Table 7:** Top 10 diseases associated with the given miRNAs predicted by CATAPULT

	hsa-mir-21		hsa-let-7a-1		hsa-mir-125b-1		hsa-mir-155
4	**151430 B-cell lymphoma 2**	7	**151430 B-cell lymphoma 2**	3	**260350 Pancreatic cancer**	4	**608232 Chronic myeloid leukemia**
7	273300 Male germ cell tumor	9	**608232 Chronic myeloid leukemia**	4	**137800 Glioma susceptibility 1**	6	151430 B-cell lymphoma 2
9	155601 Cutaneous malignant melanooma	10	273300 Male germ cell tumor	6	273300 Male germ cell tumor	8	273300 Male germ cell tumor
11	**266600 Inflammatory bowel disease 1**	13	188550 Nonmedullary Thyroid cancer 1	7	**151430 B-cell lymphoma 2**	9	155601 Cutaneous malignant melanooma
13	**608232 Chronic myeloid leukemia**	14	137800 Glioma susceptibility 1	9	155601 Cutaneous malignant melanooma	10	**137800 Glioma susceptibility 1**
14	**188550 Nonmedullary Thyroid cancer 1**	15	226150 Enterocolitis	10	114500 Colorectal cancer	12	114500 Colorectal cancer
15	226150 Enterocolitis	17	**600634 Pituitary adenoma**	11	226150 Enterocolitis	13	**188550 Nonmedullary Thyroid cancer 1**
16	181500 Schizophrenia	19	605027 Non-Hodgkin Lymphoma	12	236000 Hodgkin lymphoma	14	226150 Enterocolitis
17	131440 Myeloproliferative disorder with eosinophilia	20	266600 Inflammatory bowel disease 1	13	**188550 Nonmedullary Thyroid cancer 1**	15	158350 Cowden syndrome 1
18	605027 Non-Hodgkin Lymphoma	21	268210 Rhabdomyosarcoma	14	266600 Inflammatory bowel disease 1	16	600634 Pituitary adenoma

Know associations are omitted and records verified are in bold. The first column indicates their original rankings

**Table 8:** Top 10 diseases associated with the given miRNAs predicted by HSMP

	hsa-mir-21		hsa-let-7a-1		hsa-mir-125b-1		hsa-mir-155
3	155601 Cutaneous malignant melanooma	5	**608232 Chronic myeloid leukemia**	3	266600 Inflammatory bowel disease 1	3	**137800 Glioma susceptibility 1**
4	**608232 Chronic myeloid leukemia**	8	**151430 B-cell lymphoma 2**	5	**137800 Glioma susceptibility 1**	4	273300 Male germ cell tumor
5	**151430 B-cell lymphoma 2**	9	**600634 Pituitary adenoma**	6	273300 Male germ cell tumor	5	**608232 Chronic myeloid leukemia**
6	151400 Leukemia	11	181500 Schizophrenia	7	**188550 Nonmedullary Thyroid cancer 1**	7	**188550 Nonmedullary Thyroid cancer 1**
8	**188550 Nonmedullary Thyroid cancer 1**	12	131440 Myeloproliferative disorder with eosinophilia	9	**260350 Pancreatic cancer**	10	256700 Neuroblastoma
9	**145500 Hypertension**	14	**155255 Medulloblastoma**	10	181500 Schizophrenia	11	155255 Medulloblastoma
11	137580 Tourette syndrome	16	236000 Hodgkin lymphoma	11	**151430 B-cell lymphoma 2**	12	**155601 Cutaneous malignant melanooma**
14	273300 Male germ cell tumor	17	**176807 Prostate cancer**	12	**608232 Chronic myeloid leukemia**	13	174050 Polycystic liver disease 1
15	**256700 Neuroblastoma**	18	268210 Rhabdomyosarcoma	13	158350 Cowden syndrome 1	14	137580 Tourette syndrome
16	131440 Myeloproliferative disorder with eosinophilia	19	192600 Cardiomyopathy	14	600634 Pituitary adenoma	15	125853 Diabetes type 2

Know associations are omitted and records verified are in bold. The first column indicates their original rankings

**Table 9:** Top 10 diseases associated with the given miRNAs predicted by HSSVM

	hsa-mir-21		hsa-let-7a-1		hsa-mir-125b-1		hsa-mir-155
3	**608232 Chronic myeloid leukemia**	6	**600634 Pituitary adenoma**	4	114500 Colorectal cancer	3	**188550 Nonmedullary Thyroid cancer 1**
4	155601 Cutaneous malignant melanooma	8	**608232 Chronic myeloid leukemia**	5	266600 Inflammatory bowel disease 1	5	**137800 Glioma susceptibility 1**
5	**145500 Hypertension**	9	**155255 Medulloblastoma**	6	145500 Hypertension	6	256700 Neuroblastoma
7	**151430 B-cell lymphoma 2**	11	131440 Myeloproliferative disorder with eosinophilia	7	601626 Acute myeloid leukemia	8	**608232 Chronic myeloid leukemia**
8	**266600 Inflammatory bowel disease 1**	13	608232 Chronic myeloid leukemia	9	226150 Enterocolitis	9	273300 Male germ cell tumor
10	**188550 Nonmedullary Thyroid cancer 1**	14	268210 Rhabdomyosarcoma	10	**137800 Glioma susceptibility 1**	11	**601626 Acute myeloid leukemia**
12	601665 Obesity	15	**151430 B-cell lymphoma 2**	11	268210 Rhabdomyosarcoma	12	125853 Diabetes type 2
13	273300 Male germ cell tumor	16	150699 Uterine leiomyoma	12	273300 Male germ cell tumor	13	**114500 Colorectal cancer**
14	607464 Thyroid carcinoma	18	**176807 Prostate cancer**	13	600634 Pituitary adenoma	14	600634 Pituitary adenoma
15	247640 Lymphoblastic leukemia	19	**256700 Neuroblastoma**	14	266600 Inflammatory bowel disease 1	15	158350 Cowden syndrome 1

Know associations are omitted and records verified are in bold. The first column indicates their original rankings

There exist considerable overlap in the predictions from CATAPULT (Table 7) among these four miRNAs. Male germ cell tumor (273300) occurs within the top three predicted candidate diseases for whole four miRNAs. Nonmedullary Thyroid cancer 1(188550) and Enterocolitis (226150) also occur in all four lists. This is because CATAPULT is biased towards nodes with larger degrees and therefore may neglect important connections that are special to a single miRNA.

There exist lower degree of overlap in the top-ranked predictions returned by HSMP (Table 8) and HSSVM (Table 9) in contrast to CATAPULT. In these two tables associations verified by miRNet are in bold, from which we can discover the number of confirmed associations are 5, 5, 5, 4 and 5, 6, 1, 5 respectively, fewer than that predicted by HeteWalk, which are 8, 7, 6, 7.

## Conclusion

In this paper, we propose a heterogeneous network embedding method to predict disease associations accurately. We construct a heterogeneous network from various biological databases and obtain a representation vector for each entity in the network based on meta path [35] controlled random walk in our method. Moreover, we innovatively consider the edge weights during the representation learning and provide a *random walk-based measure* to assist in selecting meta path. The learned network embedding well captures the semantic characteristics and topological structures of the network to achieve accurate prediction of disease-related associations. Experimental results on real-world datasets shows the superiority of our method by multiple evaluations.

As for future work, we plan to combine more heterogeneous network data to improve the performance of association prediction and also generalize our HeteWalk for different genres of heterogeneous networks.

## Data Availability

The datasets of our paper are available in their websites of databases.

## Abbreviations

AUROC: Area under Receiver Operating Characteristic curve
D: disease
G: gene
GCNs: Graph Convolutional Networks
M: miRNA
SGD: Stochastic Gradient Descent
